# Efficacy of Electroconvulsive Therapy for Neuropathic Pain Comorbid with Major Depression

**DOI:** 10.1155/2020/8818553

**Published:** 2020-12-05

**Authors:** Masashi Ueda, Yuki Konishi, Kohei Sakurai, Atsuko Ikenouchi, Reiji Yoshimura

**Affiliations:** Department of Psychiatry, University of Occupational and Environmental Health, 1-1 Iseigaoka, Yahatanishi, Kitakyushu, Fukuoka 807-8555, Japan

## Abstract

We report a case of a 41-year-old male with postinjury neuropathic pain comorbid with major depression in which electroconvulsive therapy (ECT) was effective in relieving both neuropathic pain and major depression. A total of 12 sessions of bilateral ECT were performed using a Thymatron® (Somatics LLC; Lake Bluff, IL). After ECT, the patient was subsequently maintained on paroxetine, eszopiclone (2 mg/day), and alprazolam. There was no relapse for at least one year after the last ECT. This case indicates that ECT might be an alternative treatment for major depression associated with chronic neuropathic pain after traumatic injury.

## 1. Introduction

Robust evidence supports the use of electroconvulsive therapy (ECT) in several psychiatric conditions, such as major depression, bipolar disorder, and schizophrenia [1]. It has also been used for chronic pain with or without depressive symptoms [2]. A recent report indicated that ECT is effective for somatoform disorders or somatic-symptom-related disorders [3]. Deleterious changes in injured neurons associated with nociceptive receptors and descending modulatory pathways in the central nervous system can cause neuropathic pain. Neuropathic pain has high comorbidity rates for psychiatric disorders such as major depression or somatoform disorder. Moreover, neuropathic pain tends to be more refractory to conventional analgesics, such as nonsteroidal anti-inflammatory drugs and opioids [4]. It has been reported that tricyclic antidepressants (TCAs) and serotonin norepinephrine reuptake inhibitors (SNRIs) are effective for neuropathic pain [5]. It is difficult to say if the response rate for treatment of neuropathic pain by antidepressants is high enough. A case who had chronic intractable neuropathic pain successfully treated with ECT was reported [6]. We herein report a case of postinjury neuropathic pain comorbid with major depression. In this case, ECT was effective in relieving both neuropathic pain after traumatic injury and symptoms of major depression. To the best of our knowledge, this is the first report that ECT could be effective for both neuropathic pain and depressive symptoms referred to pain.

## 2. Case Presentation

The case was a 41-year-old married male without any medical histories whose right forearm was caught in a conveyor belt at a factory 2 years prior while at work. Subsequently, he underwent a total of six surgeries on his right forearm, but he had residual numbness and pain in the first finger of his right hand and decreased grip strength in his right hand. The orthopedic surgeon evaluated him for an open fracture of the right forearm and traumatic neuropathic pain. The pain led to insomnia and depressive symptoms such as irritability and fatigue. The patient had not been prescribed any drugs for the pain. Therefore, the patient was referred to our hospital. Paroxetine (37.5 mg/day) and eszopiclone (2 mg/day) were prescribed for his depressive state and insomnia. Nevertheless, the treatment did not improve the pain or the associated depressive state. ECT was introduced for pain and depression because the patient desired the treatment. Eszopiclone was stopped 3 days before ECT but paroxetine was continued. The Hamilton Rating Scale for depression (Ham-D) was 17 at this point. The patient's pain in the right forearm was rated a 10/10 on the Numeric Rating Scale (NRS), which was the most severe. A total of 12 sessions of bilateral ECT were performed using a Thymatron® (Somatics LLC; Lake Bluff, IL). The validity of the seizures was determined by brain monitoring and looking at motor spasm activity in the extremities with a tourniquet wrapped around the limb prior to succinylcholine injection. Propofol and succinylcholine for muscle relaxation were used for anesthesia. The pain and depressive state gradually improved with the concomitant recovery of strength in the right hand with treatment. The Ham-D score was reduced to 6, and the NRS score decreased to 3 after the last ECT. After ECT, the patient was subsequently maintained with paroxetine (37.5 mg/day), eszopiclone (2 mg/day), and alprazolam (0.4 mg/day), because the efficacy of ECT has been reported relatively transient [7]. He has not experienced a relapse for at least one year after the last ECT. Time courses of Ham-D and NRS scores and grip strength are shown in Table 1.

Ham-D: Hamilton Rating Scale for Depression; NRS: Numeric Rating Scale.

## 3. Discussion

We have described a case of neuropathic pain after traumatic injury comorbid with major depression. In the present case, ECT was effective in treating both neuropathic pain and depressive symptoms. Moreover, it is interesting that the motor function of the right hand was also recovered after ECT. Since TCAs and SNRIs were not tried in the present case, it was unknown if this case was pharmacotherapy-resistant. The precise mechanisms in ECT for improving pain or major depression remain unknown. Several studies using functional magnetic resonance imaging suggest that the primary action site of ECT for neuropathic pain might be the thalamus, which might play a role in noxious pain [8]. On the other hand, a meta-analysis demonstrated that the volumes of the hippocampus, amygdala, and subgenual cingulate gyrus were predictive for a favorable ECT response [9]. Thus, ECT might affect many molecules and neurocircuits and may subsequently cause morphological change in the brain. It was noteworthy that motor function recovery was associated with pain relief in this case. In this case, we suggest that ECT relieved the pain and subsequently lightened the motor restriction in his right forearm. However, we cannot conclude whether ECT directly relieved neuropathic pain or if it indirectly improved neuropathic pain via ameliorating depressive symptoms. In conclusion, ECT can be an alternative treatment for major depression associated with refractory neuropathic pain after traumatic injury. Accumulating cases and randomized control studies should be performed to confirm the efficacy of this treatment.

## 4. Conclusion

ECT was effective in treating both neuropathic pain and depressive symptoms. The precise mechanisms by which ECT improves pain and major depression are still unknown.

## Figures and Tables

**Table 1:** The course of symptoms after ECT.

Day	Number of ECT	Ham-D	NRS	Grip strength (kg)
Pre-ECT	0	17	10	14
5	3	10	7	21
9	4	8	5	22
19	9	6	3	25
27	12		3	30
1 year after		6	3	17
